# Competition between cubic and tetragonal phases in all-*d*-metal Heusler alloys, *X*_2−*x*_Mn_1+*x*_V (*X* = Pd, Ni, Pt, Ag, Au, Ir, Co; *x* = 1, 0): a new potential direction of the Heusler family

**DOI:** 10.1107/S2052252519004007

**Published:** 2019-04-24

**Authors:** Yilin Han, Mengxin Wu, Yu Feng, Zhenxiang Cheng, Tingting Lin, Tie Yang, Rabah Khenata, Xiaotian Wang

**Affiliations:** a School of Physical Science and Technology, Southwest University, Chongqing 400715, People’s Republic of China; b School of Physics and Electronic Engineering, Jiangsu Normal University, Xuzhou 221116, People’s Republic of China; c Institute for Superconducting and Electronic Materials (ISEM), University of Wollongong, Wollongong 2500, Australia; d Institute of Materials Science, Technische Universtät Darmstadt, Darmstadt 64287, Germany; e Laboratoire de Physique Quantique de la Matière et de Modélisation Mathématique (LPQ3M), Université de Mascara, Mascara 29000, Algeria

**Keywords:** all-*d*-metal Heusler alloys, tetragonal deformation, computational modelling, density functional theory

## Abstract

In this work, a series of all-*d*-metal Heusler alloys, *X*_2 − *x*_Mn_1 + *x*_V (*X* = Pd, Ni, Pt, Ag, Au, Ir, Co; *x*; = 1, 0), were predicted by first principles. The series can be roughly divided into two categories: *X*Mn_2_V (Mn-rich type) and *X*_2_MnV (Mn-poor type). Using optimized structural analysis, it is shown that the ground state of these all-*d*-metal Heusler alloys does not fully meet the site-preference rule for classic full-Heusler alloys. All the Mn-rich type alloys tend to form the L2_1_ structure, where the two Mn atoms prefer to occupy the A (0, 0, 0) and C (0.5, 0.5, 0.5) Wyckoff sites, whereas for the Mn-poor-type alloys, some are stable with XA structures and some are not. The *c*/*a* ratio was also changed while maintaining the volume the same as in the cubic state to investigate the possible tetragonal transformation of these alloys. The Mn-rich Heusler alloys have strong cubic resistance; however, all the Mn-poor alloys prefer to have a tetragonal state instead of a cubic phase through tetragonal transformations. The origin of the tetragonal state and the competition between the cubic and tetragonal phases in Mn-poor alloys are discussed in detail. Results show that broader and shallower density-of-states structures at or in the vicinity of the Fermi level lower the total energy and stabilize the tetragonal phases of *X*_2_MnV (*X* = Pd, Ni, Pt, Ag, Au, Ir, Co). Furthermore, the lack of virtual frequency in the phonon spectra confirms the stability of the tetragonal states of these Mn-poor all-*d*-metal Heusler alloys. This work provides relevant experimental guidance in the search for possible martensitic Heusler alloys in all-*d*-metal materials with less Mn and new spintronic and magnetic intelligent materials among all-*d*-metal Heusler alloys.

## Abstract 2

Changes in the *c*/*a* ratio and the effect of uniform strain for the tetragonal transformation of *X*_2 − *x*_Mn_1 + *x*_V (*X* = Pd, Ni, Pt, Ag, Au, Ir, Co; *x* = 1, 0) were studied. Surprisingly, all the Mn-poor alloys undergo possible tetragonal distortion and attain a stable tetragonal phase, whereas Mn-rich alloys do not have, or have only to a small extent, tetragonal distortion.

## 1. Introduction   

Heusler alloys have been a research hotspot for more than 100 years, gaining the attention of researchers due to their excellent properties and wide range of applications. High Curie temperatures (*T*_C_), tunable electronic structure, suitable lattice constants for semiconductors and various magnetic properties (Manna *et al.*, 2018 ▸) make Heusler alloys ideal materials for spin-gapless semiconductors (Wang *et al.*, 2018 ▸; Bainsla *et al.*, 2015 ▸; Gao *et al.*, 2019 ▸), half-metallic materials (Shigeta *et al.*, 2018 ▸; Han *et al.*, 2019 ▸; Khandy *et al.*, 2018 ▸) and shape memory alloys (Yu *et al.*, 2015 ▸; Odaira *et al.*, 2018 ▸; Li *et al.*, 2018*a* ▸,*b* ▸; Carpenter & Howard, 2018 ▸). Normally, there are three types of Heusler alloys: half-Heusler-type *XYZ* (Makongo *et al.*, 2011 ▸; Anand *et al.*, 2018 ▸; Zhang *et al.*, 2016 ▸; Hou *et al.*, 2015 ▸), full-Heusler-type *X*_2_ *YZ* (Akriche *et al.*, 2017 ▸; Babiker *et al.*, 2017 ▸; Li *et al.*, 2018*a* ▸,*b* ▸) and the equiatomic quaternary Heusler *XYMZ* materials (Bahramian & Ahmadian, 2017 ▸; Qin *et al.*, 2017 ▸; Wang *et al.*, 2017 ▸; Feng *et al.*, 2018 ▸) with stoichiometry 1:1:1:1, where the *X*, *Y* and *M* atoms are usually transition-metal atoms, whereas the *Z* atom is a main-group element. However, some new Heusler alloys have emerged, adding novel theoretical and experimental findings to Heusler’s research. As DO_3_-type *X*_3_ *Z* (Liu *et al.*, 2018 ▸) and C1_b_-type *X*_2_ *Z* (Wang *et al.*, 2016 ▸) alloys are converted from full-Heusler *X*_2_ *YZ* and half-Heusler *XYZ* alloys, all-*d*-metal Heusler alloys (Wei *et al.*, 2015 ▸, 2016 ▸; Han *et al.*, 2018 ▸; Ni *et al.*, 2019 ▸), whose atoms are entirely transition-metal elements, have created new potential for Heusler alloys. Although some all-*d*-metal Heusler alloys like Zn_2_AuAg and Zn_2_CuAu (Muldawer, 1966 ▸; Murakami *et al.*, 1980 ▸) have been studied earlier, their nonmagnetic structures limited their applications in many magnetic fields for shape memory effects. Recently, Wei *et al.* (2015 ▸) synthesized a new all-*d*-metal Heusler system Ni_50_Mn_50 − *y*_Ti_*y*_, and what is more, a possible martensitic transformation could be observed in Co-doped Ni–Mn–Ti phases. Wei *et al.* (2016 ▸) also synthesized Mn_50_Ni_40 − *x*_Co_*x*_Ti_10_ (*x* = 8 or 9.5) all-*d*-metal Heusler systems and magneto-structural martensitic transformations can be observed near room temperature. Based on this experimental work (Wei *et al.*, 2016 ▸), a very detailed theoretical study on understanding the magnetic structural transition in the all-*d*-metal Heusler alloy Mn_2_Ni_1.25_Co_0.25_Ti_0.5_ has been carried out by Ni and coworkers (Ni *et al.*, 2019 ▸). We must note, however, that research on this aspect is very rare. Recently, some interesting work brought to our attention by Tan *et al.* (2019 ▸) reported that all-*d*-metal alloys may not satisfy the site preference rule as do most classic full-Heusler alloys. Therefore, searching for new magnetic all-*d*-metal Heusler alloys and investigating their site occupation is necessary.

Examining recent studies of Heusler alloys, researchers emphasized the cubic state over the tetragonal phase, which limits progress in finding better tetragonal Heusler alloys. However, tetragonal phases are more likely to demonstrate large perpendicular magnetic anisotropy than the cubic state – the key to spin-transfer torque devices (Balke *et al.*, 2007 ▸). Additionally, tetragonal states have large magneto-crystalline anisotropy (Salazar *et al.*, 2018 ▸; Matsushita *et al.*, 2017 ▸), large intrinsic exchange-bias behaviour (Felser *et al.*, 2013 ▸; Nayak *et al.*, 2012 ▸) and a high Curie temperature. To better apply Heusler alloys to actual fields, it is also important to study their tetragonal state and the competition between cubic and tetragonal states.

Based on the above information, in this work we focused on a series of all-*d*-metal Heusler alloys, *X*_2 − *x*_Mn_1 + *x*_V (*X* = Pd, Ni, Pt, Ag, Au, Ir, Co; *x* = 1, 0). Our goals were to further strengthen the study of all-*d*-metal Heusler alloys and investigate their magnetic properties, electronic structures and site preference via first principles. We provide an in-depth discussion of their tetragonal transformations to find a stable tetragonal phase in the search for better applications in spintronics. We also explain and prove the stability of the tetragonal phases with the help of density of states (DOS) and phonon spectra.

## 2. Computational methods   

Under the framework of density functional theory (Becke, 1993 ▸), with the help of *CASTEP* code, we conducted first-principle band computations using the plane-wave pseudo-potential method (Troullier & Martins, 1991 ▸). To describe the interaction between electron-exchange-related energy and the nucleus and valence electrons, the Perdew–Burke–Ernzerhof function of the generalized gradient approximation (Perdew *et al.*, 1996 ▸; Hernández-Haro *et al.*, 2019 ▸) and ultra-soft (Al-Douri *et al.*, 2008 ▸) pseudo-potential were used, respectively. We employed a 450 eV cut-off energy, a Monkhorst–Pack 12 × 12 × 12 grid for the cubic structure and a 12 × 12 × 15 grid for the tetragonal structure of the first Brillouin region. The self-consistent field tolerance was 10^–6^ eV. The phonon energy calculation of Mn-poor type *X*_2_MnV (*X* = Pd, Ni, Pt, Ag, Au, Ir, Co) was performed in Nano Academic Device Calculator (Nanodcal) code (Taylor *et al.*, 2001 ▸).

## 3. Results and discussion   

### 3.1. Site preference and magnetism of cubic all-*d*-metal Heusler alloys, *X*_2 − *x*_Mn_1 + *x*_V (*X* = Pd, Ni, Pt, Ag, Au, Ir, Co; *x* = 1, 0)   

**Figure 1:**
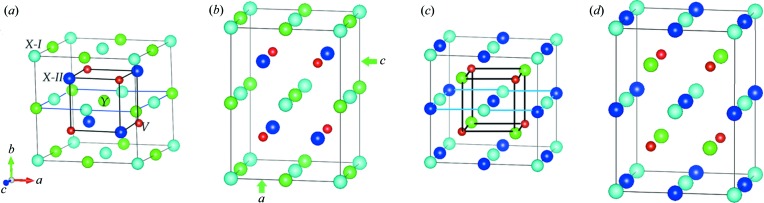
Crystal structures of (*a*) inverse cubic Heusler *X*_2_ *Y*V, (*b*) inverse tetragonal Heusler *X*_2_ *Y*V, (*c*) regular cubic Heusler *X*_2_ *Y*V and (*d*) regular tetragonal Heusler *X*_2_ *Y*V.

The site-preference rule (Luo *et al.*, 2016 ▸; Ma *et al.*, 2017 ▸; Wei *et al.*, 2017 ▸) for classic full-Heusler *X*_2_ *YZ* alloys provides fundamental guidance for their theoretical design and study of properties. When the *X* atoms carry the most valence electrons, *X* tends to occupy the A (0, 0, 0) and C (0.5, 0.5, 0.5) Wyckoff sites, and *Y* atoms, having relatively less valence electrons, prefer the B site (0.25, 0.25, 0.25). The *Z* atoms, having the least valence electrons, tend to be located at the D site (0.75, 0.75, 0.75), forming the L2_1_ type structure [or Cu_2_MnAl type, with space group 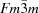 (No. 225)] as shown in Fig. 1 ▸(*c*). Another situation occurs when *Y* has the most valence electrons; the XA type [or the Hg_2_CuTi/inverse type, with space group 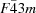 (No. 216)] is usually formed [see Fig. 1 ▸(*a*)]. The full-Heusler alloys consist of both transition-metal elements and main-group elements; however, the situation is not the same as in all-*d*-metal Heusler alloys. All-*d*-metal Heusler alloys are composed entirely of transition-metal elements without main-group atoms, so they do not necessarily conform to the site-preference rule. The desired properties depend strongly on a highly ordered structure. Hence, it is essential to study the site occupation of these all-*d*-metal Heusler alloys of *X*_2 − *x*_Mn_1 + *x*_V (*X* = Pd, Ni, Pt, Ag, Au, Ir, Co; *x* = 1, 0).

**Figure 2:**
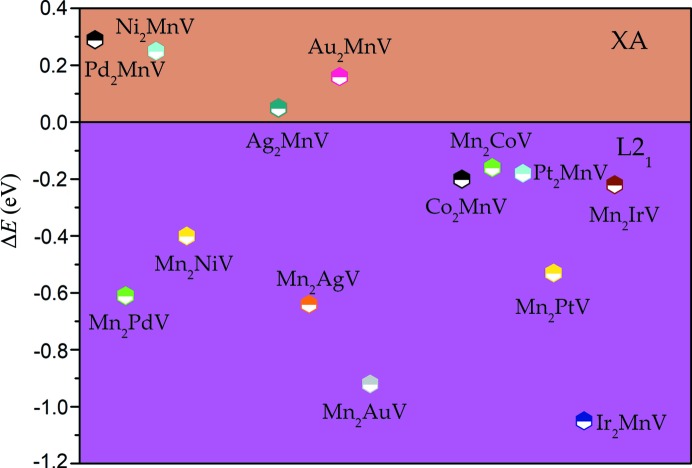
The difference in total energy of cubic-type *X*_2 − *x*_Mn_1 + *x*_V (*X* = Pd, Ni, Pt, Ag, Au, Ir, Co; *x* = 1, 0).

Given the above two site occupations, we computed Δ*E* = *E*(L2_1_) − *E*(XA) (eV per cell) of all these *X*_2 − *x*_Mn_1 + *x*_V Heusler alloys and the results are shown in Fig. 2 ▸. If Δ*E* > 0, the total energy of the L2_1_-type is more than that of XA, indicating that the XA state is more stable than the L2_1_ state. Another situation is the L2_1_ type. Fig. 2 ▸ shows that there are four alloys exhibiting XA-stable states: Ni_2_MnV, Au_2_MnV, Pd_2_MnV and Ag_2_MnV, whereas the rest of *X*_2 − *x*_Mn_1 + *x*_V are L2_1_-type. However, when the total energy difference between XA and L2_1_ phases is quite small, the two states may co-exist. So, Ag_2_MnV is hard to separate into two states, whereas Mn_2_AuV and Ir_2_MnV can be separated more easily into the L2_1_ state due to the largest |Δ*E*| (>0.8 eV), as also outlined in Table 1 ▸.

Now we discuss the application of the site-preference rule in *X*_2 − *x*_Mn_1 + *x*_V all-*d*-metal Heusler alloys. For all *X*_2 − *x*_Mn_1 + *x*_V alloys, *X* carries more valence electrons than Mn and V, so the Mn-poor type should form the L2_1_ state. *X* atoms tend toward the A and C sites, and Mn prefers the B sites. The Mn-rich alloys should be XA-type: two Mn atoms occupy the A and B sites according to the site-preference rule. However, in our calculations, the Mn-rich alloys fully disobey the site-preference rule, and some Mn-poor types meet the rule whereas others do not, suggesting that the site-preference rule does not apply to all of the all-*d*-metal Heusler alloys.

Finally, we come to study the magnetic properties of these alloys in the cubic phase; the total magnetic moments of these all-*d*-metal Heusler alloys are shown in Table 1 ▸. Mn provides the mainly magnetic moments both in XA-type and L2_1_-type, and the magnetic moments of two Mn atoms in Mn-rich alloys are always identical due to the fact that the surrounding environments of the two Mn atoms are the same in the L2_1_ phases.

### 3.2. Tetragonal transformations in all-*d*-metal Heusler alloys, *X*_2_MnV (*X* = Pd, Ni, Pt, Ag, Au, Ir, Co)   

**Figure 3:**
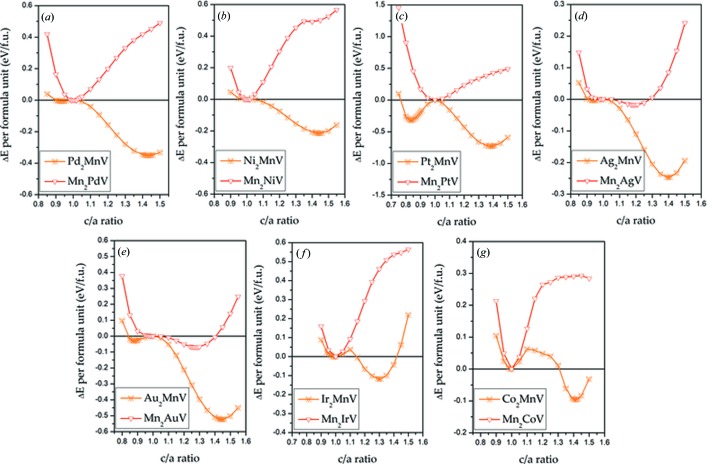
(*a*)–(*g*) Relationship between the total energy difference Δ*E* = *E*(*c*/*a*) − *E*(*c*/*a* = 1.0) and the *c*/*a* ratio for *X*_2 − *x*_Mn_1 + *x*_V (*X* = Pd, Ni, Pt, Ag, Au, Ir, Co; *x* = 1, 0).

In Fig. 3 ▸, the competition between the cubic and tetragonal phases in all-*d*-metal Heusler alloys *X*_2 − x_Mn_1 + x_V (*X* = Pd, Ni, Pt, Ag, Au, Ir, Co; *x* = 1, 0) was exhibited. We maintained the volume at the same value as in the cubic ground state and simultaneously regulated the *c*/*a* ratio to search for a stable tetragonal state. Two types of tetragonal structures, *i.e.* inverse tetragonal Heusler *X*_2_ *Y*V and regular tetragonal Heusler *X*_2_ *Y*V can be found in Figs. 1 ▸(*b*) and 1 ▸(*d*). For certain *X* elements, Mn-rich and Mn-poor types exhibit different cubic resistances to tetragonal distortion. All the Mn-poor *X*_2_MnV (*X* = Pd, Ni, Pt, Ag, Au, Ir, Co) all-*d*-metal Heusler alloys have possible tetragonal transformations, obtaining points with lower total energies, which may be a possible martensitic phase. Conversely, most of the Mn-rich alloys do not have tetragonal deformation or too small a degree of tetragonal distortion to attain stable tetragonal phases due to their strong cubic resistance.

**Figure 4:**
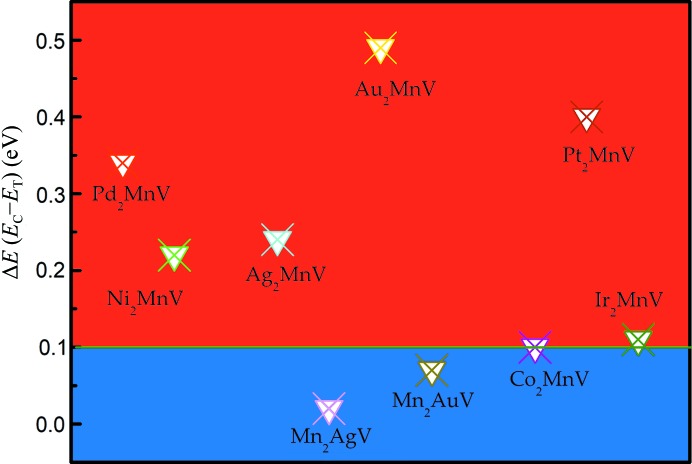
Δ*E* = *E*_C_ − *E*_T_ per formula unit as a function of *X*_2 − *x*_Mn_1 + *x*_V.

To further study the tetragonal transformation of different *X* elements, we calculated the Δ*E* = *E*(cubic) − *E*(tetragonal) (eV per cell) for all the Mn-poor *X*_2_MnV (*X* = Pd, Ni, Pt, Ag, Au, Ir, Co) structures and two Mn-rich structures (Mn_2_AgV and Mn_2_AuV) with tetragonal deformation (see Fig. 4 ▸). However, we found that although the two Mn-rich alloys have relatively lower energy states compared with the cubic state, the degree of the tetragonal distortion is too small (Δ*E* < 0.1 eV) (Wu *et al.*, 2019 ▸) to obtain a stable phase. The larger the value of Δ*E*, the easier tetragonal distortion occurs. Notably, the value of Δ*E* of Au_2_MnV is 0.49 eV, more than four times the standard Mn_3_Ga and Mn_2_FeGa′ Δ*E* (Liu *et al.*, 2018 ▸) at 0.12 eV and 0.14 eV per formula unit, respectively.

**Figure 5:**
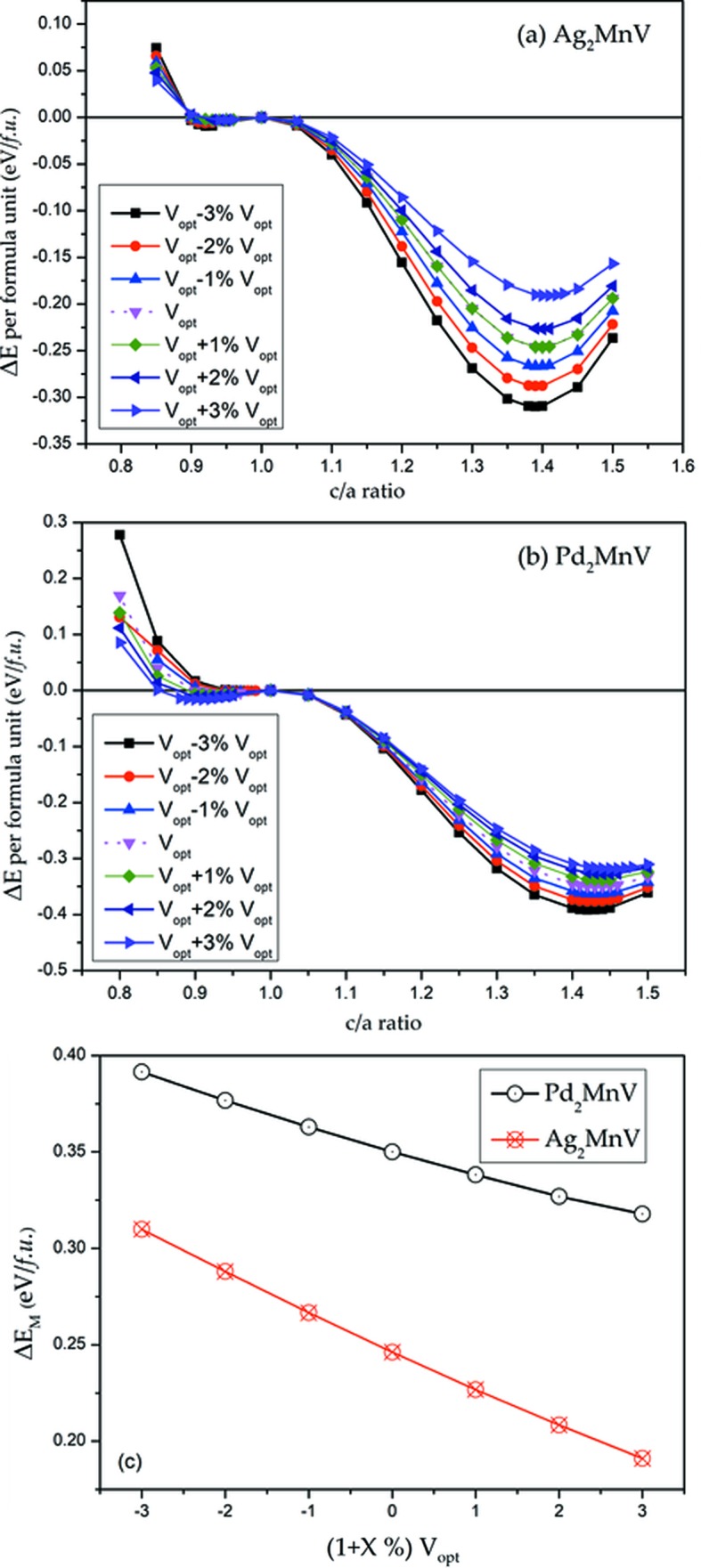
Total energy as a function of the *c*/*a* ratio for (*a*) Ag_2_MnV and (*b*) Pd_2_MnV with contraction/expansion of the unit-cell volume. (*c*) Δ*E*_M_ as functions of the *V*_opt_ + *X*%*V*_opt_ (*x* = −3, −2, −1, 0, 1, 2, 3) for Ag_2_MnV and Pd_2_MnV.

Apart from the tetragonal deformation, uniform strain should also be considered. We chose Ag_2_MnV and Pd_2_MnV as examples to study the influence of volume change. In Fig. 5 ▸, we applied values of −3, −2, −1, 0, +1, +2 and +3% of *V*_equilibrium (Opt)_ for detailed discussion. For Ag_2_MnV, the absolute value of the total energy decreases, resulting in the decline of the absolute value of Δ*E* = *E*(cubic) − *E*(tetragonal) (eV per cell) with a degree of around 0.32 to 0.18 eV per formula unit as volume expansion from V_opt_ − 3%V_opt_ to V_opt_ + 3%V_opt_, as shown in Fig. 5 ▸(*c*). Regardless of the volume changes, the possible tetragonal phases occur at *c*/*a* = 1.40. The situation is similar in Pd_2_MnV [see Fig. 5 ▸(*b*)].

### 3.3. The origin of the tetragonal state of Mn-poor *X*_2_MnV (*X* = Pd, Ni, Pt, Ag, Au, Ir, Co) alloys   

All-*d*-metal Heusler alloys are entirely composed of transition-metal elements possessing *d* states. The peak-and-valley character of the DOS in these alloys occurs due to the highly localized *d* states and the van Hove singularities at the band edges of the *d* states (Faleev *et al.*, 2017*a* ▸). The peak-and-valley character in the cubic state is one of the prerequisite conditions for *X*_2_MnV to have tetragonal distortion (the ‘smooth shift’ of DOS channels relative to *E*_F_ when adding valence electrons to the system). According to Faleev *et al.* (2017*a* ▸), the Fermi level of the cubic system is usually located at the middle of the DOS peak. However, the high DOS near *E*_F_ causes high energy, which leads to poor structural stability in the cubic state (Faleev *et al.*, 2017*a* ▸,*b* ▸; Wu *et al.*, 2019 ▸).

**Figure 6:**
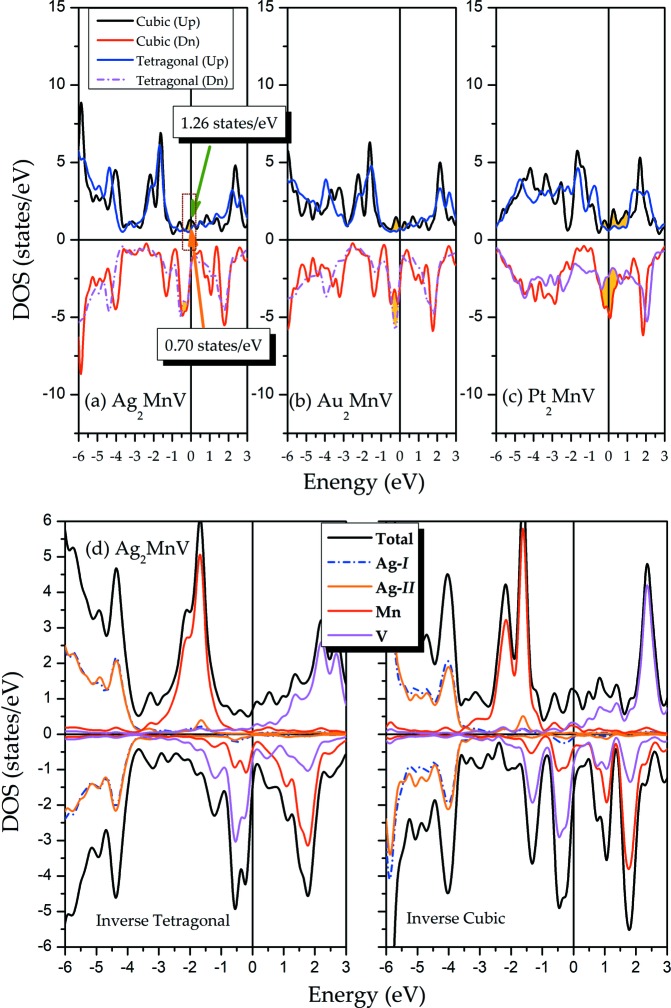
The DOS of cubic and tetragonal states for (*a*) Ag_2_MnV, (*b*) Au_2_MnV and (*c*) Pt_2_MnV. (*d*) The total and atomic DOS in inverse cubic and tetragonal states for Ag_2_MnV.

To complete an in-depth analysis of the reason for the tetragonal transformation of all-*d*-metal Heusler alloys of *X*_2_MnV (*X* = Pd, Ni, Pt, Ag, Au, Ir, Co), we selected some Mn-poor-type alloys, Ag_2_MnV, Au_2_MnV and Pt_2_MnV, as examples. We first look at Fig. 6 ▸(*a*). In the spin-up channel of Ag_2_MnV, a peak at the Fermi level changes into a valley through tetragonal deformation, with lower total energy by 0.56 states per eV. In the other channel, a high peak at around −0.5 eV is released, lowering the peak DOS at or in the vicinity of *E*_F_, which explains the stability of the tetragonal state. Similar situations can be found in Au_2_MnV [Fig. 6 ▸(*b*)] and Pt_2_MnV [Fig. 6 ▸(*c*)]. Two DOS peaks at *E*_F_ in the spin-up shift to lower energy; thus, a low energy DOS valley is located in the Fermi level after the tetragonal distortion of Au_2_MnV. Three peaks about *E*_F_ invert into a smooth valley in the spin-up channel of Pt_2_MnV in conjunction with a high peak turning into a low peak in the spin-down. We examined this to help these alloys lower the total energy then stabilize these alloys via tetragonal transformation.

Why would a high DOS (around the Fermi level) in the cubic phase become lower during tetragonal transformation? The reasons can be summarized as follows (Faleev *et al.*, 2017*a* ▸). (i) Through tetragonal distortion, the symmetry in the Brillouin zone is destroyed, which results in some *k*-points being inequivalent, causing a less peaky structure for the DOS structure. (ii) After tetragonal distortion, the symmetry of the system will be lower, and thus the degeneration of some high-symmetry *k*-points in the vicinity of Fermi level can be released. (iii) After tetragonal distortion, the bands, which are derived from the orbits that overlap in the direction of crystal contraction, become broader.

Then, we studied the total and atomic DOS of inverse cubic and tetragonal states, as shown in Fig. 6 ▸(*d*). Whether the cubic or the tetragonal structures exhibit metallic properties is explained by the definite value of the *E*_F_ in both the majority and minority of DOS. In the cubic state of Ag_2_MnV, the DOS in spin-up mainly comes from the atoms Mn and V, indicating that the total magnetic moment in the cubic phase of Ag_2_MnV is mostly contributed by Mn and V atoms. In both spin channels, the Mn and V atoms both have strong spin splitting in different directions, resulting in roughly opposite magnetic moments that cancel each other out, contributing to a small total magnetic moment (∼0.5 μ_B_) of the cubic state as shown in Table 1 ▸. After tetragonal transformation, the situation is still similar to the cubic state: the DOS of the Mn and V atoms mainly forms the TDOS structure in spin-up and spin-down channels, and the opposite spin splitting of Mn and V atoms offset each other, resulting in a small total magnetic moment (∼0.43 μ_B_). The calculated magnetic properties of tetragonal phases of these alloys have been listed in Table 2 ▸. One can see that for the regular tetragonal type, all *X* atoms have the same atomic magnetic moments due to the fact that they are in the same atomic environment, whereas for the inverse tetragonal type, the atomic magnetic moments of X-1 and X-2 are not the same.

**Figure 7:**
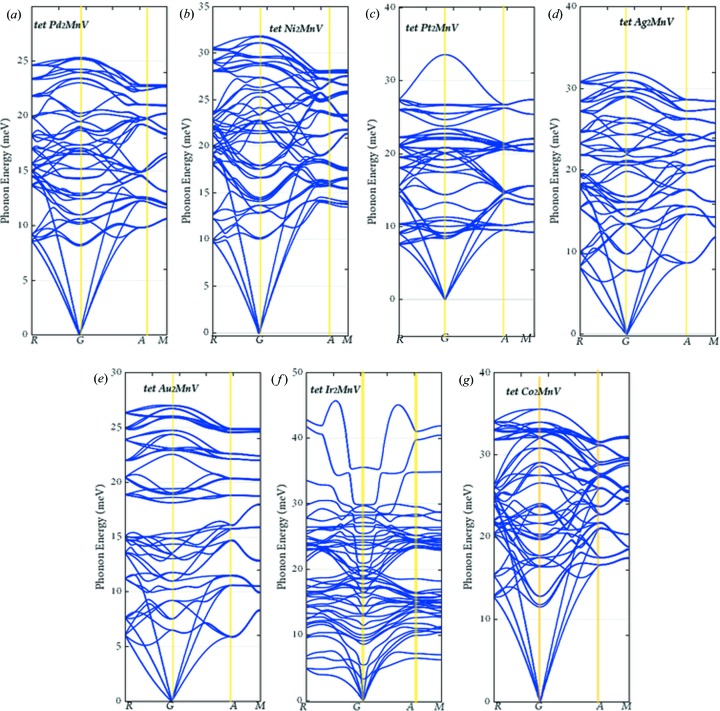
(*a*)–(*g*) Phonon dispersion curves of tetragonal *X*_2_MnV (*X* = Pd, Ni, Pt, Ag, Au, Ir, Co; *x* = 1, 0).

Finally, we introduced phonon spectra to further demonstrate the stability of seven tetragonal-type Mn-poor all-*d*-metal Heusler alloys, *X*_2_MnV (*X* = Pd, Ni, Pt, Ag, Au, Ir, Co). Unexpectedly, as shown in Fig. 7 ▸, there is no imaginary frequency in the phonon spectra of all seven alloys, verifying the stability of their tetragonal states.

## 4. Conclusions   

In this study, we highlighted a new potential direction for Heusler alloys – all-*d*-metal Heusler alloys – by investigating *X*_2 − *x*_Mn_1 + *x*_V (*X* = Pd, Ni, Pt, Ag, Au, Ir, Co; *x* = 1, 0). Firstly, we examined their atomic occupancy in the cubic phase, finding the well known site-preference rule does not apply to all of these all-*d*-metal Heusler alloys. Then, we studied changes in the *c*/*a* ratio and the effect of uniform strain for the tetragonal transformation of X_2 − x_Mn_1 + x_V. Surprisingly, all the Mn-poor alloys undergo possible tetragonal distortion and attain a stable tetragonal phase, whereas Mn-rich alloys do not have, or have only to a small extent, tetragonal distortion. Additionally, with the help of the DOS, we conducted in-depth research and provided discussion on the reasons for the transformation of the cubic phase to the tetragonal phase. Finally, we demonstrated the stability of the tetragonal state of Mn-poor all-*d*-metal alloys via the phonon spectra.

## Figures and Tables

**Table 1:** Δ*E* = *E*(L2_1_) − *E*(XA) (eV per cell), equilibrium lattice constants, total and magnetic moments in the cubic state, and the cubic stable structure

Compound *X*_2_ *YZ*	Δ*E* (eV per cell)	Structure	*a* (Å)	Mt (μ_B_ per formula unit)	MY (μ_B_)	MV (μ_B_)	MX-1 (μ_B_)	MX-2 (μ_B_)	Stable structure
Pd_2_MnV	0.29	Inverse	6.18	1.37901	3.12	−1.89	−0.04	0.2	XA
		Regular	6.11	4.52573	3.71	0.26	0.28	0.28	
Mn_2_PdV	−0.61	Inverse	5.91	4.33733	0.32	−0.03	0.98	3.07	L2_1_
		Regular	5.9	4.65193	0.52	−1.39	2.76	2.76	
Ni_2_MnV	0.25	Inverse	5.75	1.76204	2.09	−0.89	0.04	0.52	XA
		Regular	5.83	3.48349	3.21	−0.41	0.34	0.34	
Mn_2_NiV	−0.40	Inverse	5.79	2.91463	0.35	0.77	−0.84	2.64	L2_1_
		Regular	5.75	4.68429	0.91	1.09	2.43	2.43	
Ag_2_MnV	0.05	Inverse	6.22	0.51632	2.96	−2.29	−0.17	0.01	XA
		Regular	6.46	1.06416	3.75	−2.54	−0.07	−0.07	
Mn_2_AgV	−0.64	Inverse	6.25	4.05781	−0.05	−2.14	2.82	3.43	L2_1_
		Regular	6.22	4.34336	0.12	−1.98	3.10	3.10	
Au_2_MnV	0.16	Inverse	6.28	0.97787	3.15	−2.11	−0.13	0.07	XA
		Regular	6.33	4.86251	3.70	0.87	0.15	0.15	
Mn_2_AuV	−0.92	Inverse	6.02	3.69507	0.01	−1.4	1.99	3.1	L2_1_
		Regular	6.25	4.86044	0.21	−1.89	3.27	3.27	
Co_2_MnV	−0.20	Inverse	5.66	3.85062	1.83	−0.6	1.06	1.57	L2_1_
		Regular	5.69	5.68816	2.85	0.23	1.3	1.3	
Mn_2_CoV	−0.16	Inverse	5.70	3.75377	1.15	0.44	−0.26	1.15	L2_1_
		Regular	5.82	4.71505	1.46	−1.26	2.26	2.26	
Pt_2_MnV	−0.18	Inverse	6.24	1.81372	3.16	−1.58	0.0	0.23	L2_1_
		Regular	6.27	4.56093	3.72	0.23	0.31	0.31	
Mn_2_PtV	−0.53	Inverse	6.05	4.79221	0.35	−0.67	1.86	3.24	L2_1_
		Regular	5.49	4.79092	0.57	−1.4	2.81	2.81	
Ir_2_MnV	−1.05	Inverse	6.16	4.11403	3.01	−1.0	0.99	1.11	L2_1_
		Regular	6.12	5.62474	3.35	0.61	0.83	0.83	
Mn_2_IrV	−0.22	Inverse	5.96	3.78006	0.52	0.62	−0.39	3.03	L2_1_
		Regular	6.04	4.17371	0.55	−1.73	2.68	2.68	

**Table 2:** The stable tetragonal state, Δ*E* = *E*(cubic) − *E*(tetragonal) (eV per cell), *c*/*a* ratio, total and atomic magnetic moments for *X*_2_MnV (*X* = Pd, Ni, Pt, Ag, Au, Ir, Co; × = 1, *x* = 0) and *X*Mn_2_V (*X* = Ag, Au)

Compound*X*_2_ *YZ*	Stable structure	Δ*E*(eV per cell)	*c*/*a* ratio	Mt (μ_B_/formula unit)	MY (μ_B_)	MV (μ_B_)	MX-1 (μ_B_)	MX-2 (μ_B_)
Pd_2_MnV	Inverse tetragonal	0.34	1.43	1.99	3.38	−1.75	0.23	0.14
Ni_2_MnV	Inverse tetragonal	0.22	1.44	2.60	2.5	−0.71	0.38	0.43
Ag_2_MnV	Inverse tetragonal	0.24	1.40	0.43	2.97	−2.33	−0.1	−0.09
Mn_2_AgV	Regular tetragonal	0.02	1.19	4.15	0.08	−2.02	3.05	3.05
Au_2_MnV	Inverse tetragonal	0.49	1.44	3.05	−2.25	−0.09	−0.07	3.05
Mn_2_AuV	Regular tetragonal	0.07	1.28	4.25	0.08	−2.07	3.13	3.13
Co_2_MnV	Regular tetragonal	0.10	1.41	1.00	1.26	−0.28	0.01	0.01
Pt_2_MnV	Regular tetragonal	0.40	1.38	2.21	3.35	−1.53	0.19	0.19
Ir_2_MnV	Regular tetragonal	0.11	1.30	2.74	2.76	−0.16	0.07	0.07
